# Factors that influence STEM faculty use of evidence-based instructional practices: An ecological model

**DOI:** 10.1371/journal.pone.0281290

**Published:** 2023-01-31

**Authors:** Rebecca L. Sansom, Desiree M. Winters, Bryn E. St. Clair, Richard E. West, Jamie L. Jensen

**Affiliations:** 1 Department of Chemistry and Biochemistry, Brigham Young University, Provo, Utah, United States of America; 2 Department of Instructional Psychology & Technology, Brigham Young University, Provo, Utah, United States of America; 3 Department of Plant and Wildlife Sciences, Brigham Young University, Provo, Utah, United States of America; 4 Department of Biology, Brigham Young University, Provo, Utah, United States of America; University of Westminster, UNITED KINGDOM

## Abstract

Traditional teaching practices in undergraduate science, technology, engineering, and mathematics (STEM) courses have failed to support student success, causing many students to leave STEM fields and disproportionately affecting women and students of color. Although much is known about effective STEM teaching practices, many faculty continue to adhere to traditional methods, such as lecture. In this study, we investigated the factors that affect STEM faculty members’ instructional decisions about evidence-based instructional practices (EBIPs). We performed a qualitative analysis of semi-structured interviews with faculty members from the Colleges of Physical and Mathematical Sciences, Life Sciences, and Engineering who took part in a professional development program to support the use of EBIPs by STEM faculty at the university. We used an ecological model to guide our investigation and frame the results. Faculty identified a variety of personal, social, and contextual factors that influenced their instructional decision-making. Personal factors included attitudes, beliefs, and self-efficacy. Social factors included the influence of students, colleagues, and administration. Contextual factors included resources, time, and student characteristics. These factors interact with each other in meaningful ways that highlight the hyper-local social contexts that exist within departments and sub-department cultures, the importance of positive feedback from students and colleagues when implementing EBIPs, and the need for support from the administration for faculty who are in the process of changing their teaching.

## Introduction

About half of students who declare a science, technology, engineering, or mathematics (STEM) major switch or do not graduate [1]. Nearly all students (96%) who change majors cite poor teaching, with 48% citing teaching quality as a deciding factor [2]. Evidence-based instructional practices (EBIPs) improve course completion rates and raise grades in introductory college coursework, including in gateway STEM courses [3]. Types of EBIPs and the evidence supporting their use have been extensively reviewed elsewhere [4,5].

Despite increasing evidence for EBIPs, the majority of STEM faculty have not adopted these methods [6–8]. Faculty do not accurately predict which challenges they will face during EBIP implementation [9], and may not realize that challenges will evolve as their teaching practice evolves [10]. This has led to calls for research that focuses on the instructional decisions of STEM faculty members [11–13]. Faculty professional development is a high-impact practice for improving teaching [14], but there is much to be learned about the ways faculty translate professional development into classroom practice via instructional decision making.

## Conceptual and methodological frameworks

This work is guided by a conceptual framework of ecological models of human behavior [15]. Ecological models have been used extensively in public health research and interventions and are based on the levels of biological organization. These include individual humans, with unique personal characteristics; population dynamics comprising social interactions among humans; and interactions with the material world. When ecological models are used in interventions, the goal is to modify human behavior by attending to the personal, social, and contextual factors that influence human decisions. For example, in an effort to promote vaccination, personal, social, and contextual interventions are all important. Personal factors might be targeted by referring patients to reputable sources of information and balancing risks of vaccination with benefits. Social factors might be targeted by supporting non-judgmental conversations, and creating opportunities for vaccinated families to interact with anti-vaccination families. Contextual factors might be targeted with policies such as vaccine mandates within workplaces or through influence such as campaigns on social media [16]. The focus of these ecological model interventions is on changing human behavior while honoring the agency of humans operating within a complex system. One reason we chose to apply an ecological model to STEM faculty change is because it centers the agency of the faculty member to make instructional decisions and choose their teaching behaviors within the complex context of the modern university.

We also approach this work in a dual role as discipline-based educational researchers and as providers of professional development to STEM faculty. Thus, in our selection of a conceptual framework, we were looking for a model that would provide enough richness to explore the breadth and depth of possible faculty experiences, while being simple enough to apply to the design of our professional learning. Our project focused on supporting individual faculty members from across three STEM colleges with a desire to improve their teaching, and who volunteered for the program. This is in contrast to other programs which might be more directed or targeted at a specific college or department. Because of our focus on individuals, it was important to center faculty agency within the change model. Several other conceptual frameworks have been used to investigate STEM faculty change such as Teacher-Centered Systemic Reform, CACAO Model of Change, and Change Perspectives [12,17,18]. However given our focus on helping individuals change their teaching behaviors and our desire to use the conceptual framework both for research and design of the program, the ecological model was selected.

**Fig 1:**
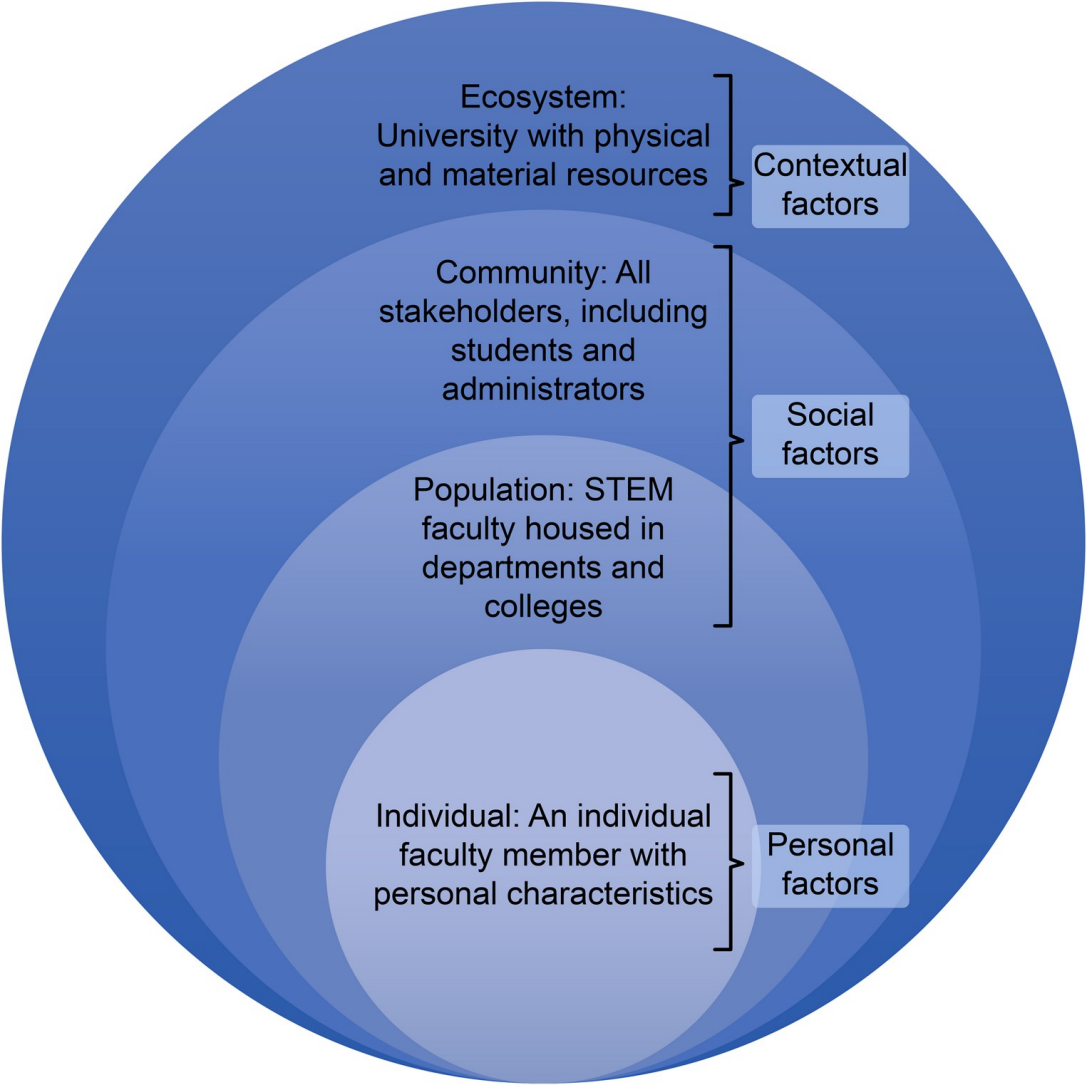
An ecological model of STEM faculty instructional decision-making. Individuals, with personal characteristics, are part of the population of STEM faculty within a department or college, who interact with other populations such as students and administrators to form the community. These living things interact with the nonliving parts of the environment, such as resources and classroom spaces, to form the ecosystem.

We framed our research by considering personal, social, and contextual characteristics that contribute to STEM faculty instructional decision-making. Fig 1 illustrates the embedded nature of humans within social and physical/material contexts. We also used this conceptual framework to design our intervention, the STEM Faculty Institute. We targeted personal factors by providing student-like experiences, direct training about EBIPs, and structured opportunities for practicing new strategies to change their attitudes, beliefs, and self-efficacy. To target social factors, we created cohort experiences where participants would share their successes and failures using EBIPs and brainstorm solutions as a group. To target contextual factors, we provided dedicated time during the workshop to create modified teaching materials and discussed how to implement EBIPs within the context of heterogeneous student preparation.

Our work was also guided by the methodological framework of hermeneutics [19]. Hermeneutics is an appropriate methodological framework for context-sensitive research environments, such as the work of faculty operating within departments and colleges and influenced by their relationships with students and colleagues [19]. This work also takes place within a historical context, when STEM reform is a priority and STEM faculty have increased awareness of EBIPs [20]. A foundational principle of hermeneutics is the idea of interpretation. The researcher is embedded within the context of the research and that embeddedness influences their interpretation of events [21]. As some of the authors of this paper and investigators on this project are STEM faculty who operate within the same departments and colleges on campus as the participants, hermeneutics was an appropriate choice of framework. The hermeneutic circle also encourages an iterative, cyclical approach to understanding the parts in the context of the whole, and the whole in the context of the parts [22]. This approach is evident in our methods as described below.

## Background

A significant effort has been made to characterize the factors that influence STEM faculty adoption and use of EBIPs. Many of these are summarized in Sturtevant and Wheeler [23]. In this section, we will consider the decision-making environments of STEM faculty members organized according to personal, social, and contextual factors that have been identified as important for STEM faculty decision making.

### Personal factors

With the exception of a few programs for graduate and postdoctoral scholars [24–26], the majority of STEM faculty begin without any training in pedagogy, much less the use of EBIPs [27], although they may bring to bear other funds of knowledge and experience [28]. In addition to a lack of pedagogical training, faculty hold strong beliefs about teaching and learning that influence their instructional choices. The conception of teaching as information delivery is persistent in the field [29,30] and correlated with traditional instructional practices [12]. In contrast, EBIPs are associated with the attitude that teachers are facilitators for student learning [31], and instructor empathy can influence student success [32]. Faculty attitudes and beliefs impact both their willingness to adopt new strategies [33], and their enacted teaching practices [34].

### Social factors

Faculty members operate within a complex social environment, interacting with students, colleagues, and university administrators. Given the significant role of student evaluations of teaching in tenure and promotion decisions, changing instructional practices and an associated lowering of evaluation scores can be a high risk proposition for faculty [27,35,36]. Interactions with colleagues may support or hinder use of EBIPs, however it has been difficult to establish shared consensus around teaching climate within departments [37]. Collaboration with colleagues and department change agents can support instructional change [14,36,38], but socialization within professional societies may prioritize research over teaching [39]. The social interactions between STEM faculty and university administrators can also affect decisions. Most faculty believe that teaching is not highly valued as part of personnel decisions [27]. All these social interactions affect STEM faculty instructional decision-making.

### Contextual factors

STEM faculty are influenced by the resources available in their environment. Time is a limited resource, both in and out of class, and STEM faculty face pressure to cover content during limited instructional time [10,36,40]. The use of EBIPs also requires preparation time. There is an opportunity cost when faculty choose to spend time on teaching instead of research [35,41,42].

Evidence-based instruction requires different resources than traditional instruction. The lack of appropriate learning environments is frequently cited as a barrier to the use of EBIPs [36]. However, Talanquer and Pollard [43] claimed that a classroom designed for EBIPs drove change at their institution. In efforts to support STEM faculty adoption and use of EBIPs, these contextual factors must be considered.

### Research question

In this study, we addressed the research question: What factors influence STEM faculty decision-making about EBIPs?

## Methods

### Setting

This study took place at a large, private, doctoral-granting university in the western United States. The average ACT score for entering freshmen in 2018 was 29/36, and the high school grade point average for entering freshmen was about 3.85/4.0. Although the university has students from all 50 U.S. states and from more than 100 foreign countries, most students identify as Caucasian (~80–85%).

### Participants

Participants were STEM faculty participating in a rich, year-long professional development program. Fifteen full-time, tenure-track faculty members from the Colleges of Engineering, Life Sciences, and Physical and Mathematical Sciences volunteered to join this program and participate in this research project, which was approved by the institution’s Institutional Review Board. The professional development included a summer workshop on EBIPs followed by a year-long mentored implementation, during which participants used EBIPs, received mentoring from a peer, reflected on changes made, and collaborated in cohort meetings. Participants received a small stipend for their participation. Our engagement with participants also included pre- and post-interviews, one-on-one work during the summer workshop, pre- and post-classroom observations, ongoing journal entries, and many informal interactions on campus. Thus, the researchers were engaged in knowing and understanding the participants as individuals, including their social and environmental contexts.

### Data collection

Prior to the summer workshop, the first two authors conducted interviews with each of the 15 participants that explored three primary areas: (a) participant beliefs about teaching and learning (e.g., How do you think students learn best?), (b) current teaching practices (e.g., Describe a typical day in your classroom), and (c) motivation to change (e.g., What barriers keep you from using more EBIPs?). Two researchers conducted the interviews together, taking turns alternately conducting and observing.

### Data analysis

We followed guidelines for qualitative data analysis described by Miles, et al. [44]. Prior to and during the study, the primary researchers recorded evolving thoughts in a reflexive journal. Then we conducted the interviews. Immediately after the interviews, we discussed noteworthy ideas and individually recorded our thoughts in the reflexive journal. The interviews were audio recorded and transcribed verbatim.

To protect the anonymity of study participants, all quotes and descriptions use gender-neutral pronouns (*they/their* instead of *he/his* or *she/hers*). Additionally, information that could identify the specific course they teach has been removed (e.g., replacing Physical Science 100 with a general education science course). Quotes were adjusted slightly for clarity by removing stutters and speech disfluencies and expanding reductions.

Using the hermeneutic circle, we each used our thoughts about the interviews to form a sense of the whole meaning, then analyzed the transcripts sentence by sentence, asking at each sentence whether the participant had communicated something about why they made instructional decisions. In this way, the whole informed the interpretation of the parts, and the parts informed the interpretation of the whole. We organized themes within the main structure of personal, social, and contextual influences. After we analyzed the transcripts individually, we met to discuss our interpretation of the interviews and the emerging themes.

After this primary analysis, we created a codebook with themes emerging from the interviews and applied it to a separate data set (from a second cohort of participants) to ensure that we were able to capture the nuances in the data using the codes. We used a constant comparison method, actively searching for ideas that were not described by the existing codes, and where patterns emerged, we added additional codes to the codebook [45]. This revised codebook was used to analyze the data for this article, and as we analyzed, we proceeded with the same attention to ideas not previously identified. Ultimately, we developed 57 unique sub-themes and 10 themes. Finally, we selected representative passages to include in the report. Due to space, we are unable to describe all of the themes identified, but we included those that stood out as the most important factors influencing instructional decision-making.

### Trustworthiness

We used reflexive journaling, prolonged engagement over the entire course of the professional development program, and weekly peer debriefing with another member of the research team to address issues of trustworthiness. In addition this manuscript was sent to the participants for a member check, and they all agreed that their thoughts were analyzed and communicated accurately.

### Limitations

This study population is not representative of all STEM faculty at all institutions. The institution is unique as the number of undergraduates is approximately 10 times that of graduate students, influencing expectations about research and teaching within the university community. The student population at this university is also unique, as students tend to be highly academically qualified and not very racially diverse.

All of the STEM faculty members who participated in this study were volunteers with some desire to improve their teaching using EBIPs. While this may be viewed as a limitation, we chose to work with this population of willing participants because we believe that a large number of STEM faculty at a variety of institutions fall into this category. Thus, understanding the factors that influence the instructional decisions of STEM faculty who express some willingness to learn more about and use EBIPs will be an essential piece of change efforts.

## Results

Results are organized according to the ecological model. The metathemes, themes, and subthemes we identified are presented in Table 1. As each code is introduced in the narrative, it will be indicated with italics.

**Table 1:** Themes about instructional decision-making identified during the analysis of interview data.

Metathemes	Themes	Subthemes	Description
Personal factors	Beliefs about teaching and learning	Facilitator	I believe that teaching is about creating an environment in which students can learn, as evidenced by my stated beliefs or my description of typical classroom activities
		Deliverer	I believe that teaching is about communicating or delivering information to students, as evidenced by my stated beliefs or my description of typical classroom activities
		Caring	I care about my students and about being a good teacher, and I regularly make efforts to improve my teaching
	Attitudes about EBIPs	Learn more	Students learn more when I use EBIPs than when I don’t
		Engagement	EBIPs increase student engagement in class or help maintain attention of students
		Uncomfortable	EBIPs are uncomfortable or unnatural for me, they do not align with my personality and preferences
		Context	EBIPs might work in some instances, but not in my specific class because it is special or unique
	Self-efficacy	Knowledge and training	I have/have not received training in pedagogy or EBIPs
		Professional development	Actively seeking out and participating in professional learning related to teaching
		Yes, I can	A general sense of confidence—as soon as I learn what EBIPs are I will be able to do them in my class
		Management	If I’ve attempted to use an EBIP and it hasn’t gone well, it was because I didn’t do a good job facilitating it, not because the EBIP is flawed
Social factors	Colleagues	Collaboration	I have/do not have opportunities for meaningful collaboration with colleagues
		Canon	There is an established body of knowledge that must be covered in my class because that’s what everyone else does
	Students	Like	Students tend to enjoy EBIPs in class
		Prefer lecture	Students prefer lecture over active engagement
	Administration	Valued	The administration values high quality teaching
		Initiatives	The administration supports/does not support regular initiatives to improve teaching
	Student evaluations of teaching	Meet the bar	The standard for good teaching is getting high enough student ratings, if my student ratings are high enough, I shouldn’t spend any more time or effort on improvement
		Negative	When I use EBIPs, I expect it will negatively impact student ratings
		Only measure	Student ratings are the primary determinant of whether my teaching is good or bad, acceptable or unacceptable for promotion
Contextual factors	Time	In class	I have a lot of content to cover and a limited amount of class time, so I have to be careful with EBIPs that might take longer
		Out of class	Preparing to teach using EBIPs is harder and takes more time and effort than lecturing
		Balancing responsibilities	I have to balance teaching with research and citizenship and there are opportunity costs to those choices
	Resources	Materials	There are no ready-made EBIP materials or lesson plans that are appropriate for my content or course
	Student characteristics	College readiness	Students these days just aren’t as prepared for college as they used to be
		Unprepared for class	When I try to use EBIPs, students don’t come prepared for class, and then the activities I’ve planned are impossible
		Heterogeneity	Some students in my class are ready for this type of learning, but others aren’t, it’s hard to teach when they start out at such different levels

### Personal factors

STEM faculty have deeply held beliefs about teaching and learning, and about EBIPs, which influence their instructional decisions. In addition to attitudes, self-efficacy with the use of EBIPs was explored.

#### Beliefs about teaching and learning

Some beliefs expressed by participants in the study related to general beliefs about teaching and learning that affect EBIP adoption. Previous research has shown that faculty may hold beliefs along a facilitator-deliverer continuum [46]. We found that our faculty participants often struggled with these beliefs, expressing a mixture of facilitator and deliverer attitudes.

The *facilitator* code was assigned when participants expressed a desire to create classroom environments that facilitate student learning or when they described classroom activities that provided evidence that they view teaching in this way. For example, one participant said, “Students learn best the things that they have some hand in figuring out. So… I have gone more and more away from ‘I am going to lecture you and tell you stuff.’” Another participant described a classroom activity that suggested a facilitator teaching style, saying:

Is cholera caused by tainted water or tainted air?…Everybody thought it was air because it’s found in places that are stinky… . Here’s an interesting data set that was collected in the 1850s… . Does this support [air] vs. [water]?

All of the participants exhibited some degree of disposition toward facilitation, as manifest in their espoused and enacted beliefs.

The *deliverer* code indicated that the participant viewed teaching as delivering information to students. One participant explained, “I mean, that’s the goal of teaching. You take what you know and give that to the people you are teaching.” Others highlighted the efficiency and clarity of lecture, saying, “In some cases, that’s the way that knowledge has to be transmitted” and it allows you “to introduce the content so it’s clear to the student.” For some faculty, lecture is the default, even when they attempt to use a more student-centered strategy. One participant described what happened when students were asked to report to the class on journal articles, saying, “It was really a student lecturing, and then we tagged out and I lectured for the rest of the time.” Interestingly, when asked to describe a powerful learning experience from when they were students, many faculty members described a good lecture. Highlighting the sometimes-disconnect between their attitudes and behaviors, one participant acknowledged that they primarily lecture, even when students cannot keep up with the deluge of information. They said, “I would really like all of those problems to be worked live on the chalkboard. And the reason is that it slows me down enough that the students have a hope of keeping up.” That the deliverer attitude appeared so often, even though most faculty participants viewed themselves as facilitators, is evidence of the persistent struggle to move toward student-centered teaching.

Beliefs about teaching as an important endeavor were coded as *caring*. This code was assigned when participants described their own efforts to be the best teachers they could be, often manifested in spending extra time or effort to get to know students, help struggling students, and make the content relevant. One participant said plainly, “I’d like to improve… I’d like to have more skills, and more students that are learning.” Another commented that even though their student evaluations have been good, they still saw ways to improve their teaching, saying, “How [do I]… always try to say, ‘How can I do this better?’ That’s motivating for me.” Finally, several participants discussed learning students’ names and trying to build a safe community in class, where everyone could participate. One participant said, “I learn all their names in all the classes that I have so they can feel comfortable talking to me and approaching me.” Another participant described the ways that they reach out to overwhelmed students, saying they “invite students that [they] see really struggling and say, ‘Hey, I can see you’re slipping behind here; why don’t you come talk to me?’” Another participant talked about how they tailor their course to the students’ interests. Recognizing that many of their students plan to pursue careers in medicine and dentistry, this instructor said, “I spend a lot of time finding new materials… that are medicinally relevant or even dentistry related.” Each of these actions demonstrates the care with which the participants approach teaching and the effort they are willing to make to become better teachers.

#### Attitudes about EBIPs

Participants also expressed attitudes directly related to EBIPs, based on what they had seen, heard, observed, or experienced.

Faculty participants generally believed students *learn more* or engage in more *complex thinking* when taught using EBIPs. One participant said, “Engaging and active is the corner of that matrix that I think most quality learning occurs in.” Another participant, who had recently tried encouraging more student-student discussions in class noticed that “once they get to participate, we can see big improvements in learning… . When they were reading the material in the textbook, they didn’t fully understand until we discussed it.” Using EBIPs to support deeper student learning can sometimes cause students to feel uncomfortable, leading one participant to say, “I’ve had a couple student evaluations that were like, ‘I had to teach myself everything!’ And I thought, ‘Good! That’s great.’ That’s what I want.” One participant commented that they wanted to “get to the higher-level thinking skills and have time… [for] interesting problems and not superficial ones.” The expression of positive attitudes about EBIPs was most often related to their ability to help students learn more or think more deeply, which suggests that faculty are aware of some of the evidence base that supports their use.

Beyond learning more, faculty perceived EBIPs as encouraging student *engagement*, recognizing that EBIPs help students stay awake, attentive, and entertained in class by providing variety. One participant claimed that “there needs to be an active component… . There needs to be something that forces them to engage every five to 10 minutes.” They went on to describe one of the major challenges of university teaching as “learning how to manage attention spans, learning how to mix up activities to keep people interested,” joking that they’re “up tap dancing on the front counter.” Engaging students was perceived as a major benefit of EBIPs, even when faculty did not see EBIPs as a means to improve student learning.

One concern with the use of EBIPs was that they were perceived as not fitting the personality of the instructor, causing the instructor to lose control of the class or being *uncomfortable* in comparison to lecture. One participant noted that because lecture is the academic tradition of universities, “I understand the material so I’m going to stand up and expound… . We do it a lot because it’s so much easier.” Another participant explained that lecture is an easy pattern to fall into, saying, “I fall back to what I’m comfortable doing, and we love that control when you know the pace, you know the content… . You know how it will be presented, and lecturing is a really good way to do that.” Other participants felt that the EBIPs they had tried “didn’t work with [their] personality,” “screwed up [their] flow,” were “scary,” or “felt forced and awkward,” causing them to avoid using those strategies more often. The sense that EBIPs are uncomfortable may be a result of limited knowledge or lack of awareness of a variety of good strategies.

Many faculty members expressed concern that EBIPs might be appropriate in some situations but not in the *context* of their specific course or STEM content. Some participants reported that for certain material lecturing just made the most sense, saying, “There was some material… [that] was difficult enough… . I felt like I had to lecture and explain what was going on.” Others noted differences between types of classes—general education vs. majors, lower division vs. upper division, and required vs. elective. One participant said they use fewer EBIPs in upper-division courses, noting that “for an upper division class, I spend more time on concepts and less time on examples.” Another participant did the opposite, saying, “In my advanced class where I only have six students, I had more than half a mind to put all the chairs at a table and just talk about stuff and totally changed the method for that class.” Faculty also expressed divergent views about when learning happens, indicating that the real learning does not happen during the regular class time but rather as students work on problems outside of class. One participant expressed that problem-solving work is “so much more efficiently done… in homework, so I push that as much as possible to [out of class] where it is most effective.” Context-specific beliefs about EBIPs demonstrate an understanding of the limitations and affordances of the strategies.

#### Self-efficacy

An individual’s sense of their own capability to use EBIPs is encompassed by the construct of self-efficacy [47].

One factor that influenced participants’ self-efficacy with the use of EBIPs was *knowledge and training*. Some of the study participants had formal training in education. Two participants had extensive training or mentorship related to science pedagogy and active learning. One said of his experience as a graduate student:

I worked with a program… developing inquiry-based learning strategies for K-12 teachers… and it made me think about all the ways we were teaching effectively and how we could potentially improve.

The other observed his postdoctoral advisor using active learning, commenting that “my advisor there was pretty progressive in her approach to teaching… . I observed her as she tried to do more active learning in her own introductory classes.”

These examples do not apply to most participants, who received no training in pedagogy. Many described the process of learning to teach at the university as a sort of trial-by-fire experience. They commented, “You’re an expert on something, and suddenly it’s like, ‘We are going to throw you in [to the classroom].’” “You get hired here, and it’s like, ‘Teach away!’” And “It’s pretty much been learning on the job.”

Although a few participants had some required teaching training as graduate students, most described it as minimal or insignificant. For example, one participant said:

During my PhD,… you taught one lecture, you wrote the test questions, and you met with the professor a few times and kind of walked through it. So, I did that once, and that was my teaching experience before [being hired].

Faculty participants also recognized that, in the absence of training, they patterned their teaching on “what I’d observed in classes I had taken before.” Another said succinctly, “The only way I know how to teach is how I was taught, which a generation ago was pretty much a teacher lecture.” Lacking formal training, or minimal training experiences, caused faculty to rely on their experiences as students.

Although most faculty had little or no formal training in pedagogy, many were familiar with a variety of evidence-based practices, including student response systems, dialoguing strategies, group work, and the flipped classroom. They knew about these strategies because they sought *professional development* for teaching. They described reading journal articles and books about teaching, attending teaching workshops in their college and elsewhere on campus, working with consultants at the Center for Teaching and Learning, and attending new faculty programs. One commented that their “work to become a better teacher has been mostly proactive,” while another shared that they “attend [teaching workshops] whenever [they] can” and find them “extremely beneficial”.

Faculty participated in these professional development activities because they wanted a sense of accountability and dedicated time for improving their teaching. One commented that they saw participating in the professional development program as an opportunity to refine their teaching because they knew they “would not do it otherwise.” Another faculty member described their participation as “a good opportunity to kick myself in the pants and try some new, different things.” The participants in our study were proactive in seeking opportunities to think about and improve teaching.

One reason why participants seek professional development is because they believe they can learn to teach better, a sentiment we coded as *yes*, *I can*. Many participants described a willingness to try new teaching strategies, saying, “I experiment a lot,” “Every semester I try something different,” and “I have the confidence to try stuff.” In describing their process of continual reflection and improvement, one participant said, “I hope I’m open to change… willing enough to say this isn’t working, and… if this isn’t working for the students, then I have got to rethink it.” In some sense, this confidence was surprising, because they also expressed a lack of knowledge about EBIPs and pedagogy generally. Acknowledging their own unfamiliarity with EBIPs and simultaneously expressing their confidence in their ability to use new strategies, one participant said, “I am sure that there are other strategies that I could incorporate that I just haven’t ever witnessed. So, I’ll see it and say, ‘That’s a great idea!’” Participants expressed confidence that yes, they can learn and implement new teaching strategies, and this enthusiasm was not dampened by a lack of training or knowledge.

Participants were also perceptive enough to identify situations where they had attempted, unsuccessfully, to use EBIPs, failing because their *management* was insufficient. One participant said, “Sometimes when I implement it, it just doesn’t seem to go well,” elaborating, “It’s probably honestly I just didn’t implement it right.” Others commented, “It would be great if I could learn how to do it in a way that doesn’t cause chaos,” and “Sometimes it works really nicely, and sometimes it doesn’t”.

To clarify that the issue was not with the strategy itself, but rather with the instructor’s *management* of the strategy in class, other participants added, “When they’re not successful, it’s mostly either lack of planning or lack of a good explanation or ‘Oh, I didn’t know this thing would happen,’” and “I think it’s squarely on me just [not being able to] figure out how to do it.” Issues with instructor management of EBIPs also centered on ensuring that all students were actively engaged. One participant explained, “It’s hard to figure out how to make that work… and really get the students to do what you need them to do.” Classroom management while using EBIPs was universally challenging for the study participants, who worried that their ineffective management might cause students to disengage in class.

### Social factors

Participants reported expectations about teaching from colleagues, students, and administrators that fall into the social parts of the ecosystem model.

#### Colleagues

Treating STEM faculty as members of the same species, the population-level of the ecological model dictates that collegial interactions among STEM faculty should be highly influential for teaching practice.

One repeated theme in this category related to cultures of *collaboration*, or lack thereof, around teaching within departments. For some faculty, there were opportunities for collaboration with colleagues teaching the same course. One participant remarked that they have “done a fair amount of joint teaching… . So we’re doing a lot of peer-to-peer development.” In one of the colleges, peer-teaching evaluations are relatively common. One participant shared that the peer would “come to your class for two lectures and you’ll come to mine and we’ll go to lunch and talk about” the teaching to learn from each other. For other faculty members, coteaching was not possible, either because they were the only person teaching a course or because they were excluded from that opportunity. One participant remarked that they have “felt quite lonely as a faculty member here,” explaining that they have “seen other faculty members doing coteaching… . It sounded like this was a positive, constructive experience, and I’ve never had the opportunity to do that with anyone.” For others, these collaborations were limited in scope to deciding on learning objectives or content goals, rather than sharing teaching methods.

Many participants expressed that one of the reasons they wanted to participate in the professional development program was the opportunity to collaborate, indicating a lack of collaboration within their departments. One expressed that nobody in their department talked about teaching, and “with colleagues, it’s ‘your class is your class’ and you do your stuff.” One participant, who sought feedback on using EBIPs from colleagues, was strongly discouraged from trying anything new, with colleagues saying, “That’s really dumb,” and “No, that’s not even possible.” Collaboration around teaching creates cultural norms that can encourage the use of EBIPs, while a lack of collaboration can be isolating and discourage them.

Despite minimal expectations from colleagues about how to teach, there were strong expectations about what to teach, which included the *canon* of the discipline. Some departments have established curriculum committees that delineate specific learning outcomes for each course. One participant remarked that “because the learning outcomes were set up by a committee in the department, you don’t feel like you can really vary all that much”.

These expectations were more pronounced for courses intended for students majoring in the discipline, for courses that serve as prerequisites for later courses, and when the course content is perceived as required by disciplinary accreditation organizations or for graduate entrance exams. One participant explained:

We are kind of restricted by the [professional association accreditation requirements] and the [graduate entrance exam] content… . And then there’s the issue if it’s [the first semester in a two-semester sequence], you’ve got to finish a certain chapter before you pass the kids off to [the second semester course].

A sense of the established canon in the field can also be influenced by commonly used textbooks. One participant described feeling constrained by the content requirements in this way: “If you look at most biology textbooks, they sort of start at biochemistry and they end at ecosystems and there’s 37 chapters and it’s like, Oh, there’s about 37 lectures, and that’s about a chapter a day, and it sort of forces you into a structure.” Faculty participants feel a sense of responsibility to their community of colleagues and to the students to ensure that courses teach all aspects of accepted knowledge in the field.

#### Students

There is the general perception by faculty that students *like* EBIPs. Whether students enjoy an experience can be an important measure of success for faculty. When describing their past experiences using EBIPs, they said, “The students generally liked it a lot,” “The students seem to enjoy it,” and “I’ve gotten a lot of good feedback on it.” There is some indication that student enjoyment goes beyond having fun to feeling that EBIPs are more effective. One participant described a student who learned more after working through a group problem-solving activity:

I have one of those students who is not always super engaged in class. This student was like, “That was super helpful! That made so much more sense! Because at the end of the class, it was like, just multiply everything together and add them up! And now I get why we’re multiplying and adding.”

Faculty participants felt that students like EBIPs that are engaging and help them learn more.

This student expectation for EBIPs was not universal. Some faculty perceived students as *preferring lecture*, because they are accustomed to it or it requires less effort. Students are taught the norms of college life early on, so it can be challenging to teach advanced students using EBIPs. One participant said,

They’re seniors by the time we get them, and so they’ve already had a lot of exposure to college classes and they have expectations for what it’s going to be like, and so when you vary from those expectations, I think they go, ‘Hey, wait, I’m really good at this other way. Why aren’t we still doing this other way?’

Another participant explained, “They want the passive experience. I, as the authority figure, tell them what they need to know for the test. They parrot back what I told them they needed to know for the test, and I put a gold star on their forehead.” When faculty perceive that students want traditional lecture instruction, it can be challenging for them to use EBIPs.

#### Administration

Most faculty participants agreed that *teaching is valued* at the university. They said the university is “generally so supportive of teaching,” that their departments put “a decent amount of emphasis on being good teachers,” and that “teaching is highly, highly valued.” One participant commented that during the required seminar series for new faculty, “they talked a lot about teaching.” Another, when asked about how their department chair would respond if they incorporated more student-centered activities, said, “I think it would be positively viewed.”

The importance of good teaching is also communicated to faculty via *initiatives*, led by departments, colleges, and the university, that support and invest in teaching. This emphasis on good teaching was communicated primarily by faculty from one college. They noted “regular teaching seminars, almost on a monthly basis.” One further described: “Our department spends a lot of time talking about [teaching] in faculty meetings. We talk about it in our college a lot, and we have teaching-learning seminars where we discuss techniques and strategies that work.” Faculty outside of that college rarely commented on teaching initiatives outside of the professional development program in which they were participating.

#### Student evaluations of teaching

Most faculty participants acknowledged that colleagues expected them to be good teachers, as measured by the code *meeting the bar* on student evaluations of teaching (SET). One participant remarked that “departmental teaching expectations [are to] get good ratings!… When it’s all said and done, that’s how you get measured.” Another said, “The main thing that we get as far as faculty discussions is just to make sure GPAs are in a certain range and that you get good student ratings. I mean, that’s kind of it; there’s not a lot of other discussion about it.” Collegial expectations about teaching suggest a minimum standard of acceptable SET scores, often with no consideration of teaching methods or evidence-based practice.

Worries about SET can be a significant disincentive to faculty deciding to use EBIPs. Several participants described the *negative* impacts to SET when they changed their teaching methods. One, who used a just-in-time teaching approach, responsive to student questions submitted prior to class, observed, “My student ratings actually went down that semester… . Students commented on disorganization.” Another, who attempted to flip the class, said, “My student ratings were really bad that year… . I can’t necessarily get those kinds of student ratings again.” In addition to having experienced lower student ratings in response to an EBIP implementation, some participants preemptively assumed that their SET scores would go down, even though they had not yet made instructional changes. “Every time you try to slip in something that’s really different, your ratings are probably going to take a little bit of a dive.” SET are one of the primary ways that students communicate to faculty what their expectations are for the course and can be negatively affected when students do not understand EBIPs or if faculty implement EBIPs imperfectly.

An additional impact of SET was in relation to the rank-and-status requirements, especially because student ratings are essentially the *only measure* (albeit an ineffective one) of teaching quality [48]. Put simply, one assistant professor commented that they need to “get [their] scores up for when [they] go up for tenure.” Describing the outsized role of student ratings in rank-and-status decisions, another participant said, “I’ve got to have good ratings because if I don’t get good ratings, then I may not keep my job or not get my promotion.” Some participants expressed frustration at the system, saying:

I can’t stand student ratings… . Those surveys don’t judge learning, in my opinion. They judge how much they [i.e., students] like the class, how much they like the professor… . That’s the thing about student ratings—like it or not, it’s one of the few metrics we have.

The metrics used by administration to measure teaching quality show faculty what is important.

### Contextual factors

Participants identified several contextual factors that influenced their decisions about whether or when to use these strategies.

#### Time

Time is a precious commodity, both for the faculty member balancing responsibilities outside of class and the instructional time in class.

A primary concern for participants was the issue of *time in class*. One participant stated simply, “We have this limitation. We have 150 minutes a week.” Another participant elaborated:

Because I’ve got this set of things that I think are critical for all my students to know. And for me to have more active learning… what fraction am I going to have to shave off so I have this chunk of time in class to be able to do active learning?

Other participants who had attempted to use a variety of EBIPs commented on how time intensive they were. One said, “I can’t afford to spend that much time on a good activity if it’s only a small piece of what we need to do.” Another commented that they consistently underestimate the amount of class time needed for student-centered activities, saying, “I always think they’re going to take 15 minutes; they take 30.” This time pressure is largely centered around ideas about content coverage. Faculty participants shared, “You feel constrained by a big list of things we have to do,” and “It’s hard to increase this interactive part while still covering all the material.”

Even when faculty recognized that delivering more content was not effective, they still felt pressure to cover all the material. One participant said, “It’s easy to want to give them more and more and more, and they’re not learning.” Surprisingly, two participants chose, after teaching a course, to remove content to improve learning. One explained, “I got rid of some material, not because it was bad material but because I was spending too much time on it and rushing other things.” While some faculty found ways to reduce course content, the need to cover material and the limited amount of time in class were significant hurdles.

In addition to the constraints posed by limited amounts of time in class, faculty frequently noted that *time out of class* to find, create, or refine class materials or respond to student work was a challenge to the use of EBIPs. Describing past efforts to implement EBIPs, they shared, “I haven’t had the time to integrate all these things,” “It took a lot of time and effort,” and “It’s a ton of work to set that up.”

The finite number of hours in the day combined with *balancing responsibilities* of teaching, research, and citizenship meant faculty had to prioritize, sometimes choosing other activities over teaching. One participant asked:

How much time can I put into this? I teach two classes a semester typically, I have administrative obligations, and I have a really ambitious research agenda. [Our institution] kind of straddles this fence of trying to be a really good teacher and a really good researcher, and sometimes it’s hard to do both really well, so there are all these trade-offs.

Time outside of class to prepare class activities and assessments and give feedback to students, while managing a variety of other responsibilities, was a significant barrier to the adoption and use of EBIPs.

#### Resources

For participants who were just beginning to experiment with EBIPs, the most common concern was the lack of available, high-quality *materials* appropriate for their courses. One participant commented that they needed “time to develop the material. To come up with good activities that are not going to be busy work, that are going to be possible and doable, and you know will engage the student and help them understand.” Sometimes, participants were able to find appropriate resources for their courses, but often they felt they had to create the materials themselves, which required a significant investment of preparation time.

For faculty further along in the adoption and use of EBIPs, a common concern was the lack of available assessment materials that aligned with the new classroom activities they were doing, including the perception that any assessment of learning by EBIPs would take longer to grade. One participant shared, “Ideally, we do these activities and they write about them. But then I have to grade that… . I don’t have time to grade all that stuff!” Another participant explained the challenge of “trying to find that balance of meaningful assessment but not overwhelming in how much time it takes me to work through it”.

#### Student characteristics

Student characteristics, including college readiness, preparation for class, and heterogeneity, influence the instructional decisions of STEM faculty.

Some study participants felt that students are generally not *college ready*, or mature enough for college-level work, and that their lack of maturity can make it challenging to use EBIPs. They commented, “The students are coming in more and more afraid of failure,” “At some point, [they] have to transition to a self-motivated learning style,” and “I don’t know how to get them to class without being like their dad. In college, you should be able to handle your life without someone telling you how to do it”.

Faculty also found it challenging to use EBIPs when students came *unprepared for class*, having not completed reading or other preparatory assignments or having done those assignments perfunctorily. One participant described a particularly challenging group of students: “I would ask questions and they would just sit there, and they didn’t read or prepare … there would just be blank looks.” When this instructor moved to a just-in-time teaching model, asking students to submit questions about the readings before class to encourage better preparation, students performed poorly. They said, “If you require the students to submit a question, you typically get someone who just kind of dashes off something to fulfill the assignment. They don’t care”.

Finally, instructors across all three colleges struggle to adapt to *heterogeneity* within student populations—with a variety of academic backgrounds and no consensus on prerequisites for their courses. One participant described the situation in a programming-intensive course:

We have a pretty wide range of student abilities coming into the course. Some have never seen programming at all, and they’re clueless. Whatever speed I go is going to be too fast. Other students have seen this before, and they’re not necessarily engaged.

Another instructor spoke of the varied interests of their students, who come from a variety of majors, saying, “You have 50 completely different individuals, and sometimes there are things that they might share, but there are also lots of things that they do differently and think differently.” Unpreparedness, immaturity, and heterogeneity in the student population can make it challenging to target classroom activities at the right level so that all students are engaged.

## Discussion

As we sought to understand participants’ experiences, we found that an ecological model can be a useful conceptual framework for understanding the complexity of STEM faculty instructional decisions about EBIPs. This framework allowed us to understand individuals’ personal, social, and contextual factors and thus target interventions to their specific needs. It also helped to expose the complex interactions and feedback loops that influence faculty practice. Together, we can use this information to identify appropriate supports that will encourage the adoption and use of EBIPs.

### Importance of individual contexts

While we know that social factors can vary dramatically between departments [36,49], this study exposed the fact that social factors also vary dramatically among individual faculty members within the same department. Six of the participants in this study came in pairs from three departments. In all three cases, they described very different social contexts, even though they came from the same department. In Department A, two faculty participants both taught the same course. One participant described their isolation from colleagues, having never had an opportunity to coteach. The other participant described sitting in on a colleague’s class and the colleague sharing all their instructional materials. In Department B, a similar situation occurred, where one participant had worked with colleagues over several years to develop the curriculum for the freshman course for majors. The other participant said that in their area each faculty member decides what they want to do individually, and they don’t even agree on what content to teach. A third pair, from Department C, teach the same course, but one does it in relative isolation while the other regularly collaborates with a colleague to develop learning activities.

Based on these examples, it would be inappropriate to assume that all members of a department or even those faculty who teach the same course experience similar social contexts. In addition to the unique personal contexts that individuals bring to the table, including their attitudes, experiences, and self-efficacy, we should also consider the unique social contexts that influence their decision-making about EBIPs [37,50].

### Importance of positive feedback

Attitudes toward EBIPs change over time and are influenced by the experiences STEM faculty have while teaching and the feedback they receive from students and colleagues. When faculty are isolated or lack social supports that encourage the use of EBIPs, they base their decisions solely on the feedback they get from students.

In one situation, a faculty member had grown exasperated at their students’ lack of preparation for class. They decided to use a guided worksheet and make students work in groups, not because they thought it would be more effective but as a punishment for coming unprepared to class:

I would ask a few questions, and there would just be blank looks, and I just kind of got frustrated with them. I’m like, “OK, I’m just going to make you do worksheets in class if you’re not going to prepare.”… So I made a lot of my own little one-, two-sheet tutorial things where I… would force them to graph it… and explain it in a qualitative [way and] process that information. And I thought it worked really well. The people who would sit there on their phones suddenly had to talk to their neighbor. They had to pay attention, they had to interact, they had to think about the material, and the class enjoyed it too.

The positive response from students, coupled with the participant’s sense that students were learning more, changed their attitudes about EBIPs. Rather than viewing EBIPs as a punishment, they came to view EBIPs as a useful learning tool.

This example is a bit of an exception more than a rule. Every participant described at least one situation where they attempted to use an EBIP and failed to facilitate it in the most effective way. These situations were often met with negative feedback from students, which discouraged faculty from trying new strategies or continuing to use the same ones. A more typical comment was

I can totally envision a scenario where I… try and incorporate [EBIPs] this first semester, and I perceive from the students, like, “Meh.” If they’re not loving it or totally engaged in it, then am I really going to try and continue? How many semesters am I going to keep on trying this until I say, “I don’t know if this is working out that well”.

In this instance, the faculty member potentially decided to discontinue the use of EBIPs in the face of negative feedback from students and the absence of any feedback from colleagues.

One participant was discouraged after using EBIPs throughout their course and seeing student evaluation scores go down. Fortunately, they had a contact at the Center for Teaching and Learning and they reached out to get advice. The consultant was able to offer enough encouragement to convince the participant to persist:

I met with [the consultant from the CTL] and I was like, “I don’t know what I should do with this because [my student evaluations] went down.”… And he said that often happens and then they start to come back up, and… you work through these things, and they’ll come back up. So I was like, “Alright! I’m going to do it!”

Imagine that this participant had experienced the same phenomenon in isolation. They likely would not have continued using EBIPs. Faculty will receive feedback from students, but the difference between persistence and resignation may be whether they also receive feedback from supportive colleagues.

### Importance of administrative support

Consistent with the two-pronged approach of bottom-up and top-down efforts [11], we should not neglect the importance of structural changes at the university that can support high-quality teaching. For example, at our institution, SET are the primary measure of teaching quality, even though we know they do not really measure teaching quality [51]. This can create a system of double jeopardy, where a faculty member might put effort into improving their teaching, taking time away from research, and be punished with lower student evaluation scores. Thus, their sincere efforts to improve harm them on both the research and teaching fronts. One participant described it this way:

Because we are evaluated for rank and status using [student evaluations], that is a risk I take. By trying to change my teaching, it may not go super well, and then I may have this dip in my [student evaluations] for a semester, maybe two… . It’s a buy-in of my time, and then I don’t know what the reward is.

The outsized impact of student evaluations on the rank-and-status process can be buffered by institutional policies that encourage multiple measures of teaching quality and support experimentation and continuous reflection on teaching [52].

## Conclusions

In this paper, we report our findings from semi-structured interviews with 15 STEM faculty members who volunteered to participate in a professional development program supporting evidence-based instructional practices. We found that personal, social, and contextual factors all influenced faculty decision-making for what teaching methods to utilize. Personal factors included beliefs about teaching and learning, attitudes about EBIPs, and self-efficacy with the use of EBIPs. Social factors included expectations from colleagues, students, and administrators, which manifested in the complex ways faculty participants viewed student evaluations of teaching. Contextual factors included time, resources, and student characteristics.

These factors interact in multifaceted ways, and STEM faculty make instructional decisions, including whether to use EBIPs, in complex environments that encompass personal, social, and contextual factors. An ecological model for STEM faculty decision-making can help frame thinking about the factors that are most relevant to an individual faculty member’s instructional decisions, allowing targeted interventions that provide individualized support. As there is so much variation among faculty members, even within the same department, this individualized approach is a necessary consideration for any systemic change efforts. Areas of focus for change efforts should also include providing supportive, collegial environments that will provide positive feedback and encouragement, even (and especially) when students don’t provide that feedback. Institutional policies that minimize the risk of trying new teaching strategies by identifying multiple measures of teaching quality can also be supportive of faculty adoption of EBIPs.

In future studies, we will continue to explore the connection between faculty members’ personal, social, and contextual factors and their classroom practice. In particular, we plan to investigate the relationships between espoused beliefs and enacted teaching practices as measured by the COPUS [53]. In addition, we hope to share a longer case study of individual participants, following their progress throughout the program, as they have transitioned into mentorship roles, and after the program’s conclusion.

## Supporting information

S1 File Contains the interview protocol and table of all assigned codes. (DOCX)
