# Supplementary material for: Factors that influence STEM faculty use of evidence-based instructional practices: An ecological model
Source: PLoS One. 2023 Jan 31;18(1):e0281290. doi: 10.1371/journal.pone.0281290 (PMC9888702; doi:10.1371/journal.pone.0281290)
Supplement: S1 File — (DOCX) [file pone.0281290.s001.docx]

# Initial Interview with Faculty

*Reassure them that we are not evaluating their teaching, but are seeking to understand how to help them through the STEMFI program.*

**Background and Beliefs about Teaching (<15 minutes, go in order)**

1. Tell me a little bit about your teaching background. (Probe for: How long have you been teaching, in what settings/roles, time at BYU, what types of courses, etc.)
2. Tell me about a powerful/positive/effective STEM learning experience that you’ve had as a student. (Probe for: What was the teacher doing? What were you doing? What made it such a good experience?)
3. As an educator, what do you think your role should be? (Probe for: What is your responsibility in the learning process?)
4. What do you think the student’s role as the learner should be? (Probe for: What is their responsibility in the learning process? Specifically what should they do in class? Out of class?)
5. Can you describe a time when you felt successful as a teacher? (Probe for: What did you do, and what did your students do?)
6. What do you think *student-centered teaching* is, or how would you describe student-centered teaching to a colleague? How is it different from traditional instruction? (Probe for: When have you used it in the past? What have you heard about it? What do you think it’s good for? When is it appropriate to use it?) (Help them understand student-centered teaching as focusing on the learning that happens, not the teaching that happens.)

**Current Teaching Practices (<15 minutes)**

1. Please describe a typical day in your classroom. (Probe for: What are you doing? What are the students doing? For how much time/what proportion of class?)
   1. *What types of content do you normally teach via lecture? Via student-centered methods?*
2. What do you see as some of your strengths as an educator?
3. What do you see as some of your challenges as an educator?
4. Tell me about a time you decided to try something new in your class.
   1. Probe for motivations: WHY did they want to change?
   2. Probe for strategies: WHAT were you trying to change.
   3. Probe for social context: WHO did you talk to about the change?
   4. Probe for barriers: WHAT made the change difficult?

**Ecological Model of STEM Faculty Instructional Decision-Making (<15 minutes)**

1. What barriers keep you from using more student-centered approaches? What do you think would enable you to use more student-centered approaches?

*Personal Factors*

1. What changes do you hope to make in your future teaching? (Probe for student-centered teaching. Is that part of their goals?)
   1. There are pros and cons to any teaching strategy. What do you think are the pros of (your chosen strategy)? The cons?
   2. What do you think might make this change difficult?
2. How confident are you in your abilities to use student-centered strategies?

*Social Factors*

1. Tell me about the teaching culture in your department. What expectations are there around teaching? (Probe for: Expectations of your colleagues? Expectations of your students? Expectations of the department or college leadership? Disciplinary societies?)

*Environmental Factors*

1. Are there any factors outside of your control that you think influence your ability to use student-centered strategies in the classroom?

**STEMFI**

1. What are you most excited about or what do you hope to gain from your STEMFI experience?
2. What are you most worried about when you think about your participation in STEMFI?
3. How else can we help you to have a successful STEMFI experience? How can we prepare the workshop to be most effective?

| Metatheme | Theme | Subtheme | Description | Occurrence* |
| --- | --- | --- | --- | --- |
| Personal Factors | Attitudes about EBIPs | Engagement | EBIPs increase student engagement in class or help maintain attention of students | 34 |
|  |  | Comfortable (+) | EBIPs are comfortable and natural for me, they align with my personality and preferences | 18 |
|  |  | Uncomfortable (-) | EBIPs are uncomfortable or unnatural for me, they do not align with my personality and preferences | 25 |
|  |  | Complex Thinking | EBIPs facilitate deeper and more complex types of thinking that would occur otherwise | 13 |
|  |  | Context | EBIPs might work in some instances, but not in my specific class because it is special or unique | 20 |
|  |  | Doesn't Work | EBIPs are not effective for student learning, if I've tried to use an EBIP and it didn't go well, it is because the EBIP was flawed not me | 5 |
|  |  | Illegitimate | The educational research that supports the use of EBIPs is not legitimate scholarship | 2 |
|  |  | Learn More | Students learn more when I use EBIPs than when I don't | 38 |
|  |  | Traditional | Lecture is the most effective or best way to teach students what they need to know | 8 |
|  | Beliefs about Teaching and Learning | Caring | I care about my students and about being a good teacher, and I regularly make efforts to improve my teaching | 61 |
|  |  | Dissatisfaction | I am not currently satisfied with the way that I'm teaching. I know there are better ways | 19 |
|  |  | Deliverer | I believe that teaching is about communicating or delivering information to students, as evidenced by my stated beliefs or my description of typical classroom activities | 47 |
|  |  | Facilitator | I believe that teaching is about creating an environment in which students can learn, as evidenced by my stated beliefs or my description of typical classroom activities | 103 |
|  |  | Keep In | I want to help all students succeed and stay in a STEM major or career | 11 |
|  |  | Weed Out | Part of my responsibility is to serve as a gatekeeper to prevent unqualified students from progressing in a STEM major | 7 |
|  | Self-Efficacy with use of EBIPs | Facilitation | If I've attempted to use an EBIP and it hasn't gone well, it was because I didn't do a good job facilitating it, not because the EBIP is flawed | 50 |
|  |  | Failure OK (+) | I regularly experiment with new teaching strategies and they sometimes fail, but failure is how we learn | 10 |
|  |  | Failure not OK (-) | If I'm going to try something new, I need it to work on the first try because failure is unacceptable | 7 |
|  |  | Lack of Knowledge | Not knowing about EBIPs or not having ever received training in pedagogy | 38 |
|  |  | Knowledge | Having received training in pedagogy or knowing about EBIPs | 25 |
|  |  | Professional Development | Actively seeking out and participating in professional learning related to teaching | 34 |
|  |  | Yes, I Can | A general sense of confidence--as soon as I learn what EBIPs are I will be able to do them in my class | 21 |
| Social Factors | Administration | Only Ratings | Student ratings are the primary determinant of whether my teaching is good or bad, acceptable or unacceptable for promotion | 10 |
|  |  | Initiatives (+) | The administration supports regular initiatives to improve teaching | 6 |
|  |  | No Initiatives (-) | The administration does not support regular initiatives to improve teaching | 3 |
|  |  | Teaching Valued (+) | The administration values high quality teaching | 6 |
|  |  | Teaching not Valued (-) | The administration does not value high quality teaching | 1 |
|  | Colleagues | Meet the Bar | The standard for good teaching is getting high enough student ratings, if my student ratings are high enough, I shouldn't spend any more time or effort on improvement | 8 |
|  |  | Canon | There is an established body of knowledge that must be covered in my class because that's what everyone else does | 12 |
|  |  | Collaboration (+) | I have opportunities for meaningful collaboration with colleagues | 29 |
|  |  | Collaboration (-) | I do not have opportunities for meaningful collaboration with colleagues | 19 |
|  |  | Do as I'm Doing | I am encouraged to do the same things that my colleagues do, whether that is traditional or evidence-based | 12 |
|  |  | Nonconformity | I teach differently from my colleagues, which could be viewed favorably or unfavorably | 5 |
|  |  | Professional Association | Professional associations in my field encourage good teaching and/or offer professional development opportunities around teaching | 2 |
|  |  | Research First | Research should be prioritized above all other pursuits | 2 |
|  | Students | Like (+) | Students tend to enjoy EBIPs in class | 17 |
|  |  | Dislike (-) | Students tend not to enjoy EBIPs in class | 10 |
|  |  | Ratings (+) | When I use EBIPs, I expect it will positively impact student ratings | 5 |
|  |  | Ratings (-) | When I use EBIPs, I expect it will negatively impact student ratings | 14 |
|  |  | Want Lecture | Students prefer lecture over active engagement | 15 |
| Contextual Factors | Time | Balancing Responsibilities | I have to balance teaching with research and citizenship and there are opportunity costs to those choices | 12 |
|  |  | Content Coverage (Time in Class) | I have a lot of content to cover and a limited amount of class time, so I have to be careful with EBIPs that might take longer | 68 |
|  |  | Preparation time | Preparing to teach using EBIPs is harder and takes more time and effort than lecturing | 35 |
|  | Resources | Materials | There are no ready-made EBIP materials or lesson plans that are appropriate for my content or course | 26 |
|  |  | Class size | EBIPs are easier to facilitate in classes of a certain size, larger or smaller | 10 |
|  |  | Resources General | I have/do not have access to resources such as equipment, technology, the Center for Teaching and Learning, that can help me teach in innovative ways | 22 |
|  |  | Space | The classroom I use is set up/not set up in a way that is conducive to EBIP use | 3 |
|  | Student Characteristics | Unwilling Students | Students resist or refuse to participate in group work or EBIPs I try to use in class | 18 |
|  |  | Grade | Students are only concerned with their grade and not with really learning the material | 5 |
|  |  | College Readiness | Students these days just aren't as prepared for college as they used to be | 12 |
|  |  | Heterogeneity | Some students in my class are ready for this type of learning, but others aren't, it's hard to teach when they start out at such different levels | 17 |
|  |  | Student Inexperience | Students are not accustomed to using EBIPs, so it's hard to facilitate them well | 9 |
|  |  | Unprepared Students | When I try to use EBIPs, students don't come prepared for class, and then the activities I've planned are impossible | 36 |

*Occurrence is the number of excerpts that were tagged with this code out of 1045 total excerpts from 15 interview transcripts.
